# Scoring system and a simple nomogram for predicting radioiodine refractory differentiated thyroid cancer: a retrospective study

**DOI:** 10.1186/s13550-022-00917-8

**Published:** 2022-07-29

**Authors:** Ye Liu, Yuhua Wang, Wanchun Zhang

**Affiliations:** 1 Department of Nuclear Medicine, Shanxi Bethune Hospital, Shanxi Academy of Medical Sciences, Tongji Shanxi Hospital, Third Hospital of Shanxi Medical University, Taiyuan, 030032 China; 2 Tongji Hospital, Tongji Medical College, Huazhong University of Science and Technology, Wuhan, 430030 China

**Keywords:** ^18^F-fluorodeoxyglucose positron emission tomography/computed tomography, Molecular imaging, Radioiodine refractory differentiated thyroid cancer, Recurrence, Predictive factors

## Abstract

**Background:**

Differentiated thyroid carcinoma (DTC) originates from abnormal follicular cells and accounts for approximately 90–95% of thyroid malignancies. The diagnosis of radioiodine refractory DTC (RR-DTC) is based on clinical evolution and iodine uptake characteristics rather than pathological characteristics. Thus, it takes a long time to become apparent, and the definition of RR-DTC covers multiple aspects. We aimed to analyze the clinical and molecular imaging characteristics of patients with RR-DTC and identify independent predictors to develop an RR-DTC scoring system and a simple nomogram for predicting the probability of RR-DTC. We reviewed the data of 404 patients with metastatic DTC who underwent both post-RAI WB therapy scintigraphy and ^18^F-fluorodeoxyglucose (^18^F-FDG) positron emission tomography/computed tomography. Data on the clinical features and molecular characteristics of RR-DTC and non-RR-DTC cases were obtained from medical records. We screened for predictors using univariate analyses, obtained independent predictors through multivariate analyses, and then established a scoring system and a simple nomogram for predicting RR-DTC according to the corresponding odds ratio (OR) values.

**Results:**

Diagnosis at age ≥ 48 years (OR, 1.037; 95% confidence interval [CI], 1.007–1.069), recurrence between the operation and iodine-131 treatment (OR, 7.362; 95% CI 2.388–22.698), uptake of ^18^F-FDG (OR, 39.534; 95% CI 18.590–84.076), and the metastasis site (OR, 4.365; 95% CI 1.593–11.965) were highly independently associated with RR-DTC. We established a scoring system for predicting RR-DTC, showing that the area under the receiver operating characteristic curve (AUC) with a cutoff value of 10 points (AUC = 0.898) had a higher discernibility than any other single independent predictor. The risk factors of RR-DTC in nomogram modeling include diagnosis at age ≥ 48 years, recurrence between the operation and iodine-131 treatment, uptake of ^18^F-FDG, and the site of metastasis. The concordance index (c-Index) of the nomogram was 0.9.

**Conclusions:**

We demonstrated that a predictive model based on four factors has a good ability to predict RR-DTC. An index score ≥ 10 points was found to be the optimal index point for predicting RR-DTC. Moreover, this nomogram model has good predictive ability and stability. This model may help establish an active surveillance or appropriate treatment strategy for RR-DTC cases.

## Background

The incidence of thyroid cancer has been rising worldwide. According to Surveillance and Epidemiology and End Results data, there were 44,280 cases and 2200 deaths from thyroid cancer in 2021. Differentiated thyroid carcinoma (DTC) originates from abnormal follicular cells and accounts for approximately 90–95% of thyroid malignancies [1]. Multimodal treatment, including surgery, selective radioactive iodine (RAI) therapy, and thyroid-stimulating hormone (TSH) suppression therapy, can achieve good outcomes with excellent 10-year overall survival exceeding 90% and lead to survival times of more than 40 years [2, 3]. Unfortunately, the rates of local recurrence and distant metastasis are 30% and 10%, respectively [4]. There are multiple treatments, such as surgery and RAI therapy, for these patients [5]. RAI therapy is an effective treatment for patients with DTC with metastasis. With these treatments, only one-third of patients achieve a complete response; the remaining two-thirds of patients can be classified as having radioiodine refractory differentiated thyroid cancer (RR-DTC), and their overall prognosis is poor [6–8] with a median overall survival of 2.5–3.5 years [9]. The diagnosis of RR-DTC is based on clinical evolution and iodine uptake characteristics rather than pathological characteristics, so it takes a long time to become apparent, and the definition of RR-DTC covers multiple aspects. Therefore, early identification of RR-DTC is important to optimize treatment strategies during long-term follow-up [10]. Early identification of malignant/metastatic tissue with non-radioiodine avidity can avoid unnecessary radioiodine therapy. RR-DTC cases with concentrated RAI can be managed with multiple therapeutic options, including local and systemic therapy.

Most studies of RR-DTC are conducted on the prognostic factors of DTC at the time of initial diagnosis, including the patient’s age, sex, tumor characteristics, extrathyroidal spread, and clinical staging [11]. There are few predictive factor models, particularly for RR-DTC. The prediction of RAI-related factors helps understand the natural course of RR-DTC and optimize patient management. Li et al. [12] studied a multivariate prediction model for postoperative RR-DTC, but the factors in the study were limited and did not include predictions at the molecular imaging level. Recently, the roles of nuclear medicine and molecular imaging methods have attracted widespread attention in the determination of RR-DTC.

## Methods

This study aimed to analyze the clinical and molecular imaging characteristics of patients with RR-DTC and to identify independent predictors, so as to establish an effective multivariable prediction model for RR-DTC.

### Patients and data collection

Following study approval by the Ethics Review Board of Shanxi Bethune Hospital (approval number: YXLL-KY-2021-005), we reviewed clinical records to identify 404 postoperative DTC patients who had metastatic lesions. These cases were detected using various imaging examinations, including cervical ultrasound, roentgenography, computed tomography (CT), and magnetic resonance imaging and had undergone both RAI therapy and ^18^F-fluorodeoxyglucose positron emission tomography/computed tomography (^18^F-FDG PET/CT) examinations between January 2014 and January 2021. The Ethics Committee waived the requirement for informed consent because of the retrospective study design. The patients had been referred for at least two courses of RAI after total or near-total thyroidectomy. Levothyroxine sodium tablets were discontinued for approximately 3–4 weeks before RAI treatment, while a low-iodine diet was maintained. The administered activity of RAI was determined according to the patient's recurrence risk stratification, the currently found metastatic site, and the level of stimulating thyroglobulin. The individual activity range was 3.7–9.25 GBq. Patients met the requirements for inclusion in the study when the time interval between baseline ^18^F-FDG PET/CT and the initiation of treatment was < 1 week. The end-point event was defined as the presence of RR-DTC or non-RR-DTC. The Discovery Elite (General Electric Healthcare, Boston, MA, USA) was used for PET/CT examination and the Discovery 670 Elite (General Electric Healthcare) was used for whole-body iodine imaging examination. RR-DTC was diagnosed when patients had the following conditions according to the 2015 guidelines of the American Thyroid Association [2, 7]: “(1) the foci never concentrate RAI; (2) despite previous evidence of RAI concentration, the foci lose the ability to be RAI-avid; (3) despite the significant concentration of RAI, concentration is presented in some foci but not in others; or (4) metastasis progression within 1 year after RAI therapy.” Moreover, some patients with visible radioactive iodine uptake in lesions are not cured despite several treatment courses, but yet their disease does not progress according to Response Evaluation Criteria in Solid Tumors (RECIST) criteria. For these patients, the probability of obtaining a cure with further radioactive iodine treatment is low and side effects might greatly increase. Therefore, patients receiving RAI doses of more than 600 mCi may be considered to have RR-DTC [7, 8]. The follow-up period ranged between 6 and 90 months (median, 20 months), and to date, no patient in the cohort has died because of thyroid carcinoma. The data on the RR-DTC and non-RR-DTC clinical features, such as sex, age, operation frequency, histological subtype, recurrence risk group, extrathyroidal extension, autoimmune thyroid disease, tumor diameter, multi-focality, lymph node involvement (including numbers and proportion of lymph node metastasis in the central or lateral neck region), locally advanced disease, clinical stage, recurrence between the operation and iodine-131 treatment, the time from the first operation to the first iodine-131 treatment, the site of metastasis, and molecular characteristics, such as the uptake of iodine-131 and the uptake of ^18^F-FDG in the first iodine-131 treatment, were acquired from the medical records. PET/CT findings were compared with those of other imaging modalities, such as ultrasound, CT, MRI, and post-RAI whole-body therapy scintigraphy. PET/CT findings were considered positive when visually perceptible and discrete FDG uptake against background activity was noted and the maximum standardized uptake value (SUVmax) was ≥ 2.5 points. PET/CT examination results were interpreted by at least two experienced nuclear medicine diagnosticians, and a consensus was achieved for all patients.

### Statistical analysis

The t-test was used for the comparison of normally distributed data between the groups, the Wilcoxon rank-sum test was used for the comparison of non-normally distributed quantitative data between the groups, and the Chi-squared or Fisher's exact test was used for the comparison of categorical data between the groups. The factors related to RR-DTC were screened through univariate analyses. Significant variables selected in the univariate analyses were included in multivariate logistic regression. Odds ratios (ORs) and 95% confidence intervals (CIs) were calculated to determine the correlation of all potential predictors. Through multifactor logistic regression analysis, the characteristics of independent factors were assigned different scores according to the ORs, and a scoring system was established. We evaluated the optimal cutoff values by using receiver operating characteristic (ROC) curves for converting skewed variables into categorical variables. After the initial univariate analysis, a forward selection method was used to build the multivariate logistic model. Inclusion and exclusion of variables were determined using the likelihood ratio and LR method, with *P* = 0.05 for inclusion and *P* = 0.1 for exclusion. A nomogram was established to predict the risk of RR-DTC in patients, and a calibration curve was drawn using bootstrap (B = 100) to evaluate the performance of the nomogram prediction model. Box and scatter plots were used to compare mean scores and show the distribution of scores. Then, we identified the best points with high sensitivity and low false negative rates (1-specificity). *P*-values < 0.05 were considered statistically significant, and the analyses were performed using SPSS software (version 26.0; IBM Corp., Armonk, NY, USA) and the "rms" package of R (R Foundation for Statistical Computing, Vienna, Austria).

## Results

### Characteristics of the patients

**Fig. 1:**
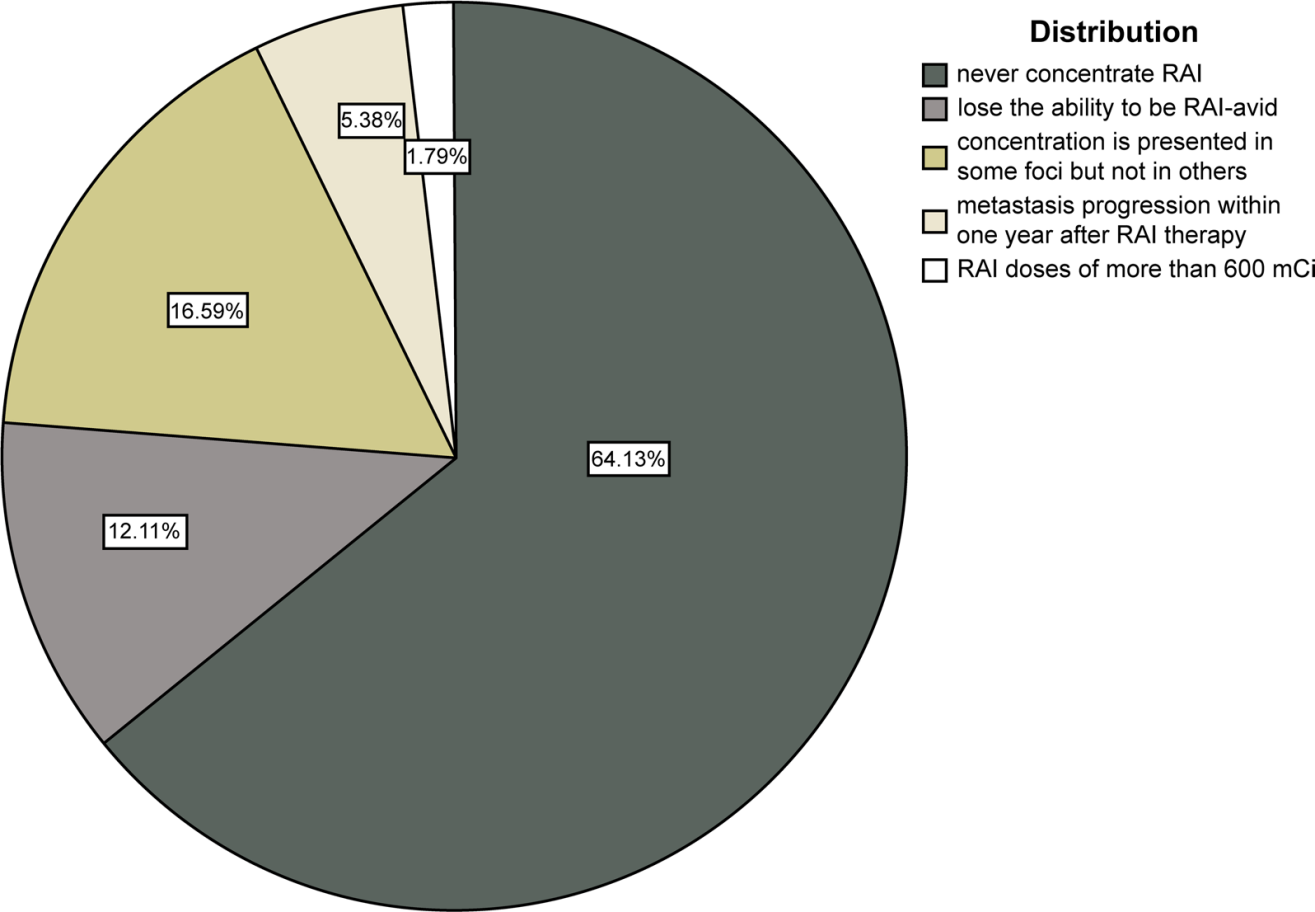
Distribution of RR-DTC. 1. The foci never concentrate RAI; 2. Despite previous evidence of RAI concentration, the foci lose the ability to be RAI-avid; 3. Despite the significant concentration of RAI, concentration is presented in some foci but not in others; 4. Metastasis progression within 1 year after RAI therapy; 5. RAI doses of > 600 mCi. Patients who meet the characteristics of types 1–4 are defined as having the corresponding RR-DTC type, and those who do not meet the criteria of types 1–4 are defined as having RR-DTC type 5. RAI, radioactive iodine; RR-DTC, radioiodine refractory differentiated thyroid carcinoma

**Fig. 2:**
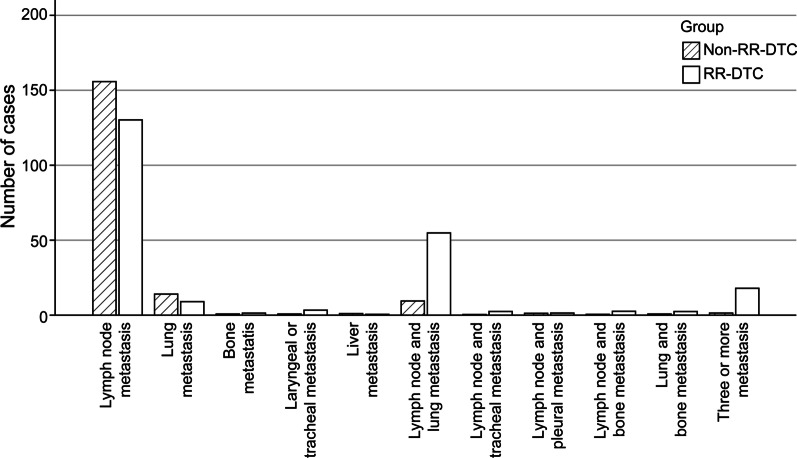
Distribution of metastatic sites. The metastatic sites were divided into 11 groups: 1. Lymph node metastasis; 2. Lung metastasis; 3. Bone metastasis; 4. Laryngeal or tracheal metastasis; 5. Liver metastasis; 6. Lymph node and lung metastasis; 7. Lymph node and tracheal metastasis; 8. Lymph node and pleural metastasis; 9. Lymph node and bone metastasis; 10. Lung and bone metastasis; and 11. Three or more metastatic sites. RR-DTC, radioiodine refractory differentiated thyroid carcinoma

Between January 2014 and January 2021, among the 1403 postoperative DTC patients treated for RAI therapy, 788 patients were diagnosed with metastatic lesions; of these, 404 patients underwent ^18^F-FDG PET/CT examination at < 1 week before or after initial iodine treatment and underwent at least two courses of radioiodine therapy. Therefore, 404 patients were enrolled in the present study. The end-point event was defined as the presence of RR-DTC or non-RR-DTC. Follow-up examinations were performed until RR-DTC or non-RR-DTC was evident. The time to determine the presence of RR-DTC ranged from 4 to 72 months (median, 6 months). We categorized patients into the RR-DTC and non-RR-DTC groups. Of the total 404 patients (mean age, 6 ± 13 years; age range, 11–77 years; male-to-female ratio,1:2.4), 223 patients (55.2%) had confirmed RR-DTC, while 181 patients (44.8%) had non-RR-DTC. The distribution of the main reason for a patient to be defined to have RR-DTC is presented in Fig. 1. The sites of metastasis involved the lymph nodes, lung, bone, larynx, trachea, pleura, liver, kidney, adrenal gland, and bone marrow (Fig. 2).

### Univariate analysis of the impact of RR-DTC

**Fig. 3:**
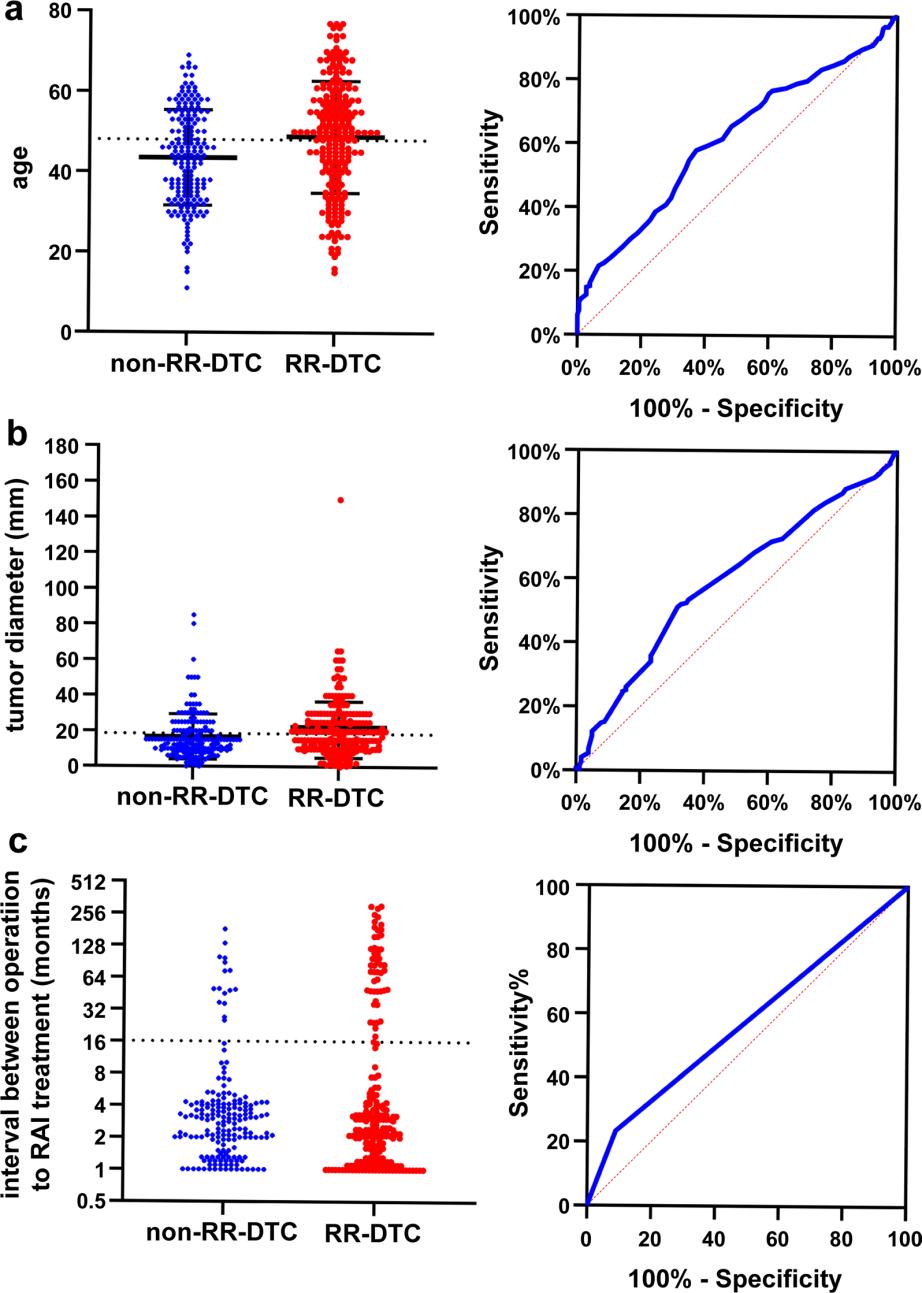
ROC curve to predict RR-DTC. **a** The optimal age at diagnosis was 48 years, and the AUC was 0.613. The sensitivity and specificity were 58% and 63%, respectively. **b** The optimal primary tumor diameter was 18.5 mm, and the AUC was 0.591. The sensitivity and specificity were 51% and 68%, respectively. **c** The optimal time from the first operation to the first iodine-131 treatment was 16 months, and the AUC was 0.572. The sensitivity and specificity were 23% and 91%, respectively. ROC, receiver operating characteristic; RR-DTC, radioiodine refractory differentiated thyroid carcinoma; AUC, area under the curve

The clinical, histopathological, and tumor characteristics of the two groups are summarized and compared in Table 1. We found that the age at diagnosis, primary tumor diameter, and the time from the first operation to the first iodine-131 treatment were significantly different between the RR-DTC and non-RR-DTC groups. For these four continuous variables, we used cutoff values to convert them into categorical variables. To determine the optimal cutoff values, we used the ROC curve, which allowed us to predict RR-DTC in terms of age at diagnosis, primary tumor diameter, and the time from the first operation to the first iodine-131 treatment. These cutoff values were 48 years of age (area under the curve [AUC] = 0.613), 18.5 mm (AUC = 0.591), and 16 months (AUC = 0.572), respectively (Fig. 3a–c). In univariate analysis, the following nine factors were found to significantly increase the risk of RR-DTC by comparing RR-DTC with non-RR-DTC patients: age at diagnosis ≥ 48 years (*P* < 0.001), operation frequency (*P* < 0.001), autoimmune thyroid disease (*P* = 0.011), primary tumor diameter > 18.5 mm (*P* = 0.007), clinical stage (*P* < 0.001), recurrence between the operation and iodine-131 treatment (*P* < 0.001), the time from the first operation to the first iodine-131 treatment (*P* = 0.001), the uptake of ^18^F-FDG (*P* < 0.001), and the site of metastasis (*P* < 0.001).

**Table 1:** Patient demographics, tumor histology, and laboratory results

	RR-DTC	Non-RR-DTC	*P*-value
	223	181	
Sex (male/female)	70/153	50/131	0.410
Age (mean ± SD, years)	48.79 ± 13.93	43.49 ± 11.88	0.001*
≥ 48/ < 48	129/94	67/114	0.001*
Operation frequency (once/two or more)	162/61	159/22	0.001*
Histological subtype (PTC/FTC/Mixed)	208/6/8	179/2/0	0.722
Recurrence risk group (low, medium/high risk)	26/197	24/157	0.627
Extrathyroidal extension (no/pathological/intraoperative/both)	117/39/40/27	113/28/26/14	0.203
Autoimmune thyroid disease (no/yes)	187/36	133/48	0.011*
Tumor diameter (mean ± SD, mm)	2.08 ± 1.58	1.68 ± 1.26	0.007*
≥ 18.5/ < 18.5	114/109	57/124	0.001*
Multi-focality (no/yes)	95/128	79/102	0.833
Site of lymph node metastasis (no/central/lateral neck region/both)	22/42/23/136	13/49/16/103	0.230
Numbers of lymph node metastasis (mean ± SD, number)	9.20 ± 8.16	9.02 ± 8.33	0.832
Proportion of lymph node metastasis (mean ± SD, %)	37.07 ± 0.27	40.05 ± 0.27	0.265
Locally advanced (no/yes)	190/33	164/17	0.103
Clinical stage (I/II/III/IVA/IVB)	110/63/7/0/43	135/31/10/2/3	0.001*
Recurrence between the operation and iodine-131 treatment (no/yes)	167/56	161/20	0.001*
Time from the first operation to the first iodine-131 treatment (mean ± SD, months)	25.93 ± 56.64	8.90 ± 22.66	0.001*
≥ 16/ < 16	52/171	16/165	0.001*
Site of metastasis			
(single-site/multi-partial metastases)	143/80	170/11	0.001*
(local/distant metastases)	133/90	155/26	0.001*
Uptake of ^131^I (negative/positive)	123/100	92/89	0.386
Uptake of ^18^F-FDG (negative/positive)	61/162	170/11	0.001*

^*^Statistically significant difference

RR-DTC, radioiodine refractory differentiated thyroid carcinoma; SD, standard deviation; ^18^F-FDG, ^18^F-fluorodeoxyglucose; PTC, papillary thyroid carcinoma; FTC, follicular thyroid cancer

### Independent predictors related to RR-DTC in multivariate analysis

The ORs associated with RR-DTC were determined by nine single factors included in the logistic regression model. Of the nine variables, there were no significant differences in operation frequency (OR, 0.284; 95% CI 0.079–1.016), autoimmune thyroid disease (OR, 0.631; 95% CI 0.308–1.294), primary tumor diameter ≥ 18.5 mm (OR, 1.002; 95% CI 0.788–1.274), clinical stage (OR, 0.803; 95% CI 0.340–1.894), or the time from the first operation to the first iodine-131 treatment (OR, 1.005; 95% CI 0.996–1.014). However, age at diagnosis ≥ 48 years (OR, 1.037; 95% CI 1.007–1.069), recurrence between the operation and iodine-131 treatment (OR, 7.362; 95% CI 2.388–22.698), the uptake of ^18^F-FDG (OR, 39.534; 95% CI 18.590–84.076), and the site of metastasis (OR, 4.365; 95% CI 1.593–11.965) displayed highly independent associations with RR-DTC. These results are presented in Table 2.

**Table 2:** Multivariate analyses of factors related to radioiodine refractory differentiated thyroid cancer

Variable	Odds ratio	95% CI	*P*-value
Age (≥ 48/ < 48 years)	1.037	1.007–1.069	0.015*
Operation frequency (once/twice or more)	0.284	0.079–1.016	0.053
Autoimmune thyroid disease (yes/no)	0.631	0.308–1.294	0.209
Tumor diameter (≥ 18.5/ < 18.5 mm)	1.002	0.788–1.274	0.988
Clinical stage (I/II/III/IVA/IVB)	0.803	0.340–1.894	0.616
Recurrence between the operation and iodine-131 treatment (yes/no)	7.362	2.388–22.698	0.001*
Time from the first operation to the first iodine-131 treatment (≥ 16/ < 16 months)	1.005	0.996–1.014	0.290
Site of metastasis (single-site/multi-partial metastases)	4.365	1.593–11.965	0.004*
Uptake of ^18^F-FDG (negative/positive)	39.534	18.590–84.076	0.001*

CI, confidence interval; ^18^F-FDG, ^18^F-fluorodeoxyglucose

^*^Statistically significant difference

### Scoring system for predicting RR-DTC

**Fig. 4:**
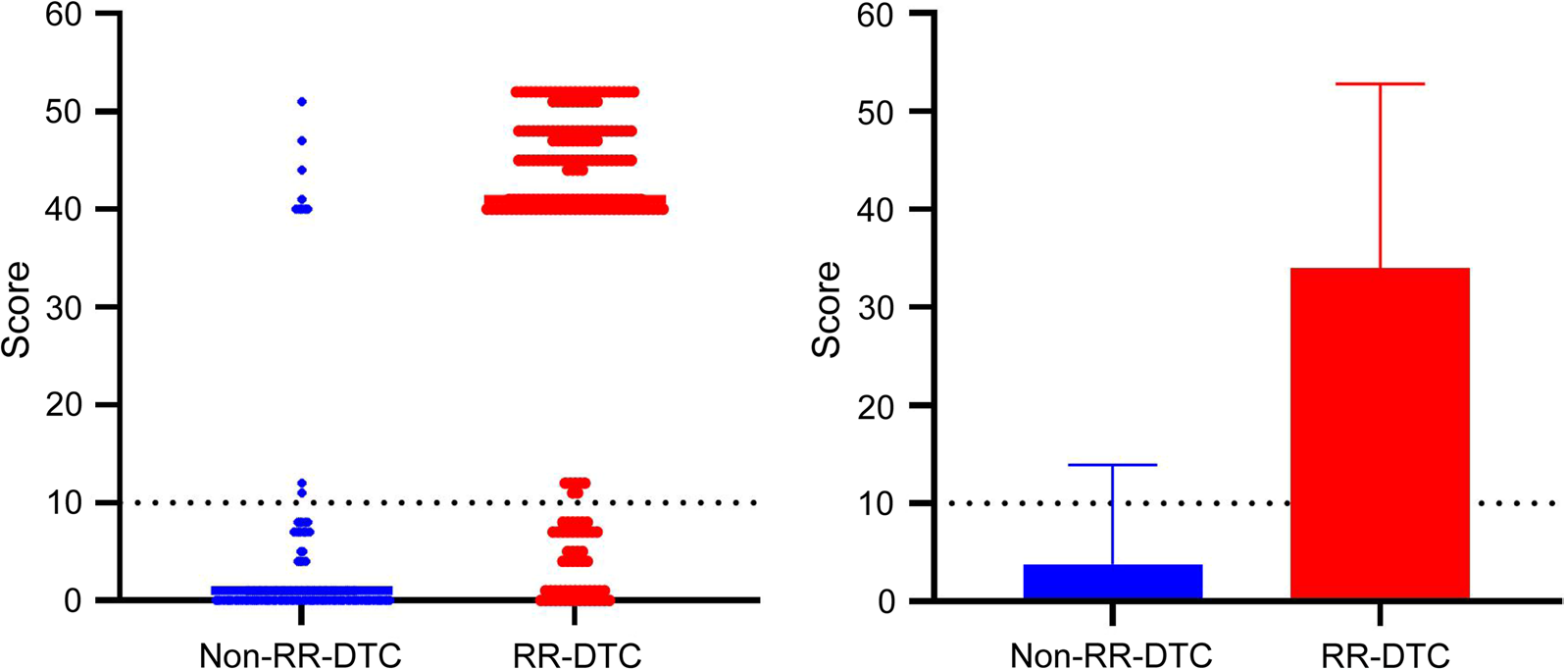
Scatter diagram of the fractional distribution in the RR-DTC and non-RR-DTC groups. The division into the groups was made according to the scoring system. There were significant differences in scores between the two groups (*P* < 0.001). ROC, receiver operating characteristic; RR-DTC, radioiodine refractory differentiated thyroid carcinoma; AUC, area under the curve

**Fig. 5:**
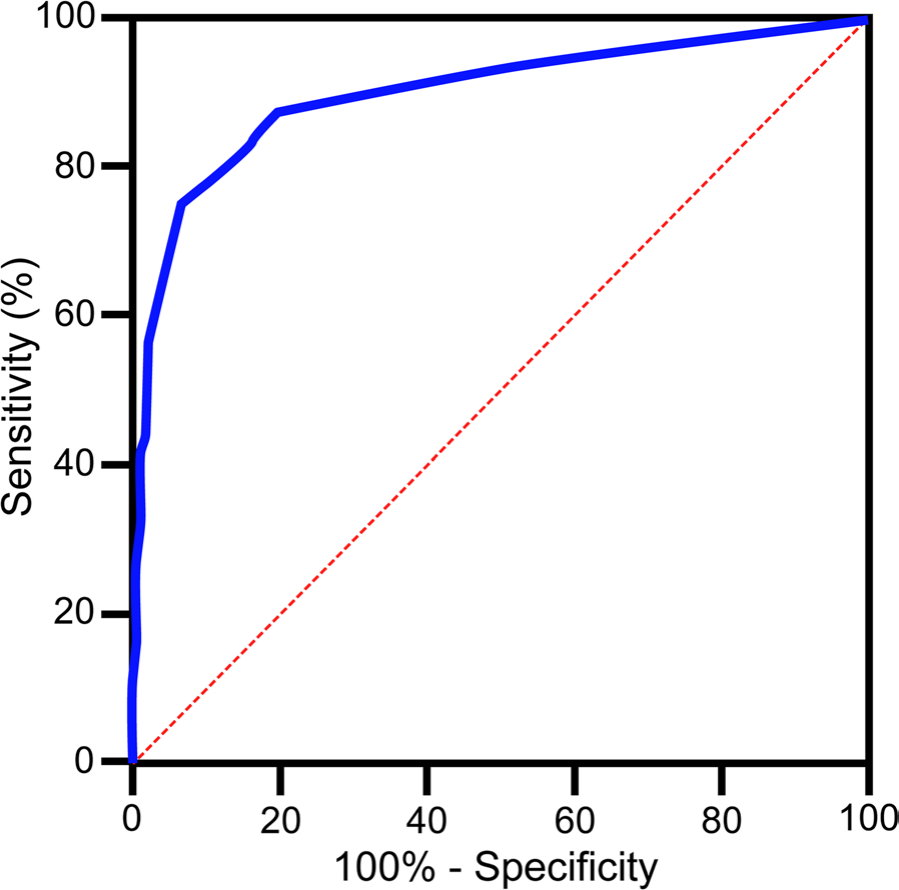
ROC curve to determine the optimal score for predicting RR-DTC. The AUC was 0.898, and a cutoff value of 10 points was found to be the best for distinguishing between RR-DTC and non-RR-DTC patients. The sensitivity and specificity were 76.0% and 93.0%, respectively. ROC, receiver operating characteristic; RR-DTC, radioiodine refractory differentiated thyroid carcinoma; AUC, area under the curve

**Fig. 6:**
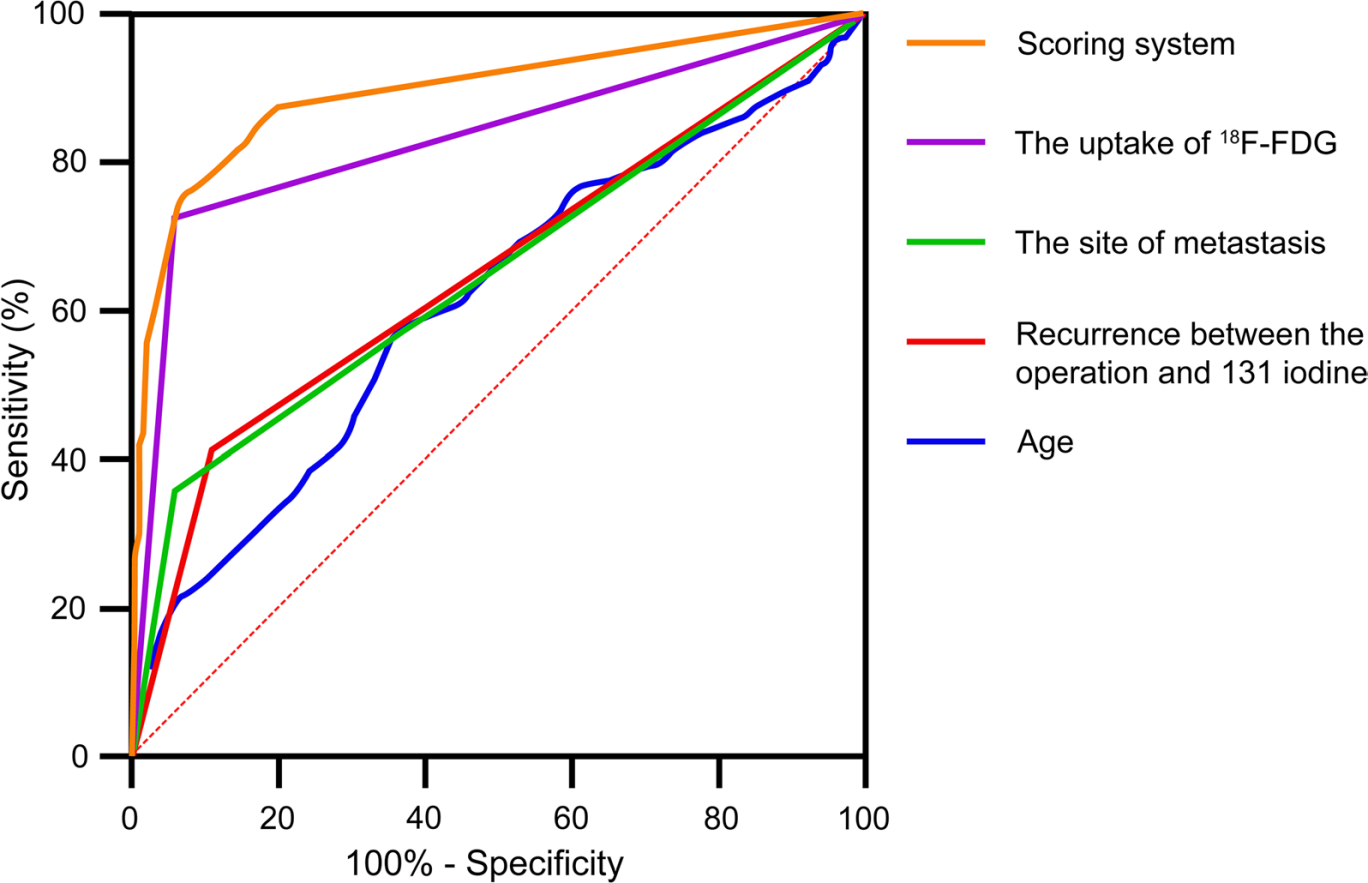
The AUC of the scoring system was larger than that of the other independent predictors. It had a higher discrimination power than other single independent predictors (largest AUC = 0.833). ^18^F-FDG, ^18^F-fluorodeoxyglucose; AUC, area under the curve

According to the OR values of the multivariate logistic regression, different score values were obtained for the characteristics related to RR-DTC. The evaluated cutoff points for each predictor are presented in Table 3: age at diagnosis ≥ 48 years, with 1 point; recurrence between the operation and iodine-131 treatment, with 7 points; multi-partial metastases, with 4 points; positive uptake of ^18^F-FDG, with 40 points. Based on this model, we calculated the scores of 404 patients. The sum of RR-DTC and non-RR-DTC points was calculated. The scatter diagram of the fractional distribution is presented in Fig. 4a. The number of high scores in the non-RR-DTC group decreased, and the number of high scores in the RR-DTC group increased. The average scores were significantly different: 33.96 ± 18.80 and 3.82 ± 10.09 points in the RR-DTC and non-RR-DTC groups, respectively (Fig. 4b). Finally, using the ROC curve we found that 10 was the optimal score for predicting RR-DTC. Its sensitivity, specificity, and Youden index values were 76.0%, 93.0%, and 0.69, respectively at 10 points. The AUC for the scoring system was 0.898 (Fig. 5). RR-DTC or non-RR-DTC were determined with 10 cutoff values. Subsequently, 169 individuals were correctly classified as RR-DTC, 168 individuals were correctly classified as non-RR-DTC, 13 individuals were misclassified as RR-DTC, and 54 individuals were misclassified as non-RR-DTC, indicating an accuracy of 83.41%. Compared with other independent predictors, as shown in Table 4, the scoring system has higher predictive value. In addition, as shown in Fig. 6, the AUC for the scoring system had a higher discernibility than any other single independent predictor.

**Table 3:** Scoring system for predicting the radioiodine refractory differentiated thyroid cancer

Variable	Odds ratio	Score
Age at diagnosis (≥ 48 years)	1.037	1
Recurrence between the operation and iodine-131 treatment (yes)	7.362	7
Site of metastasis (multi-partial metastases)	4.365	4
Uptake of ^18^F-FDG (positive)	39.534	40
		Total: 52

^18^F-FDG, ^18^F-fluorodeoxyglucose

**Table 4:** Predictive value for the scoring system and independent risk factors

Variable	Sensitivity (%)	Specificity (%)	Youden index	AUC
Age (≥ 48/ < 48 years)	58.0	63.0	0.21	0.613
Recurrence between the operation and iodine-131 treatment (no/yes)	41.0	89.0	0.30	0.651
Site of metastasis (single-site/multi-partial metastases)	36.0	94.0	0.30	0.649
Uptake of ^18^F-FDG (negative/positive)	73.0	94.0	0.67	0.833
Scoring system	76.0	93.0	0.69	0.898

AUC, area under the curve; ^18^F-FDG, ^18^F-fluorodeoxyglucose

### A simple nomogram for predicting RR-DTC

**Fig. 7:**
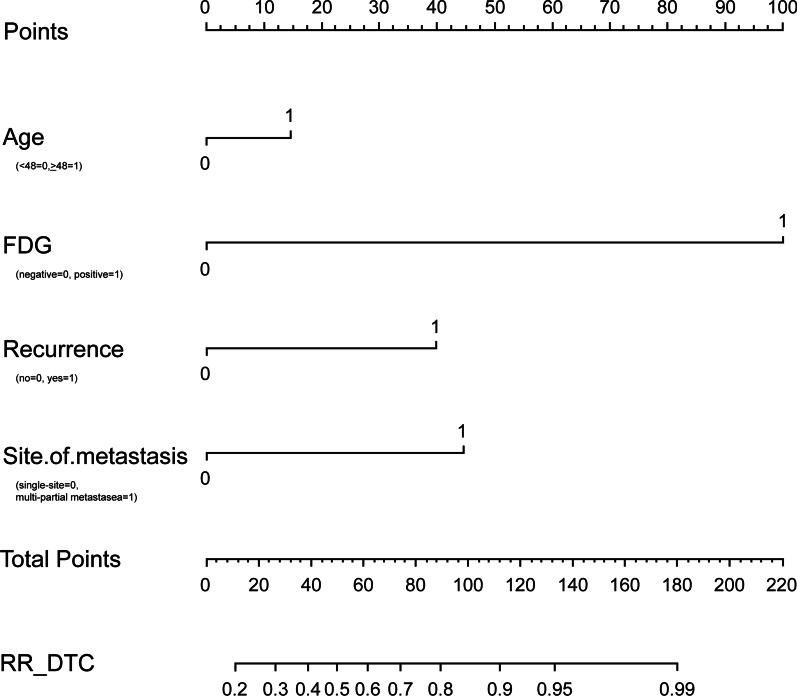
Nomogram predicting the probability of occurrence of RR-DTC. Age (0: < 48,1: ≥ 48), ^18^F-FDG (0: negative, 1: positive), recurrence between the operation and iodine-131 treatment (0: no, 1: yes), site of metastasis (0: single-site, 1: multi-partial metastases). ^18^F-FDG, ^18^F-fluorodeoxyglucose; RR-DTC, radioiodine refractory differentiated thyroid carcinoma

**Fig. 8:**
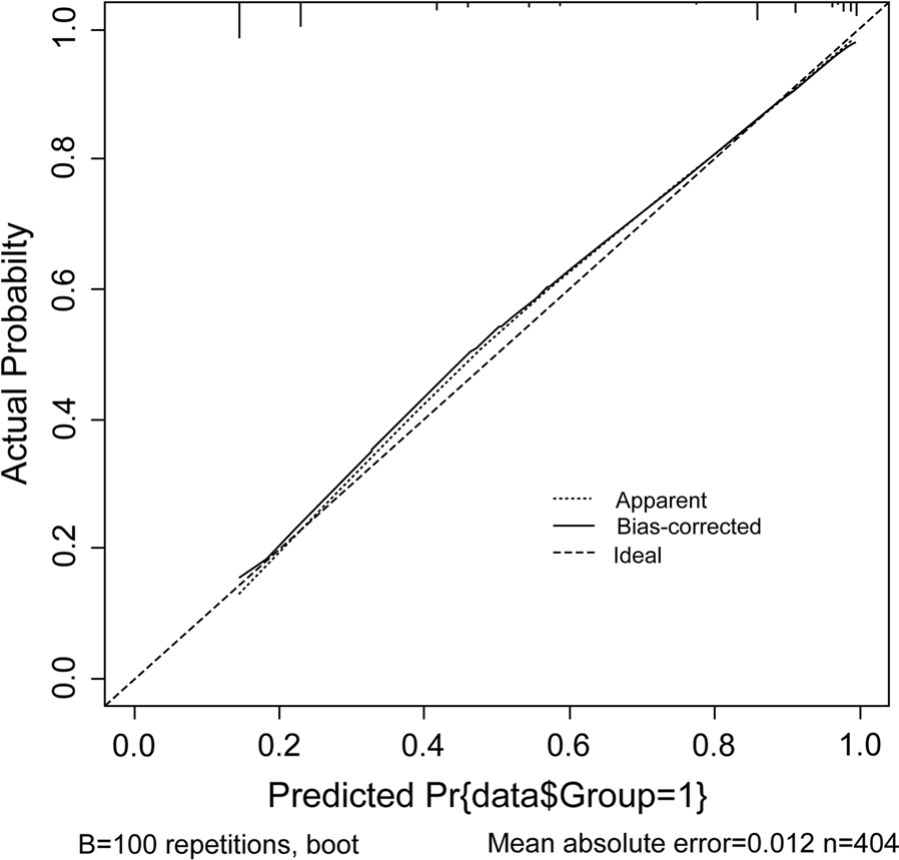
Calibration curves for predicting the RR-DTC model. RR-DTC, radioiodine refractory differentiated thyroid carcinoma

Logistic regression filters the variables and incorporates the above factors (age, recurrence between the operation and iodine-131 treatment, the uptake of ^18^F-FDG and the site of metastasis) into the nomogram modeling through R language. The nomogram is presented in Fig. 7, and the c-Index of the model is 0.9. The calibration curve is drawn in Fig. 8.

## Discussion

In the present study, we used a moderately large sample size to investigate the clinical and molecular imaging characteristics of patients with RR-DTC and the ability of these characteristics to predict RR-DTC, with the overall aim of establishing an effective multivariable prediction model for RR-DTC; such a model may prove valuable in further optimizing the treatment strategy in the early stages of metastatic DTC. Our study found that nine predictors were significantly related to RR-DTC based on univariate analyses. In addition, four independent predictors of RR-DTC were confirmed in multivariate logistic regression analysis: age at diagnosis ≥ 48 years, recurrence between the operation and iodine-131 treatment, the uptake of ^18^F-FDG, and the site of metastasis. According to the ORs, different scores were assigned to predictors that were positively correlated with RR-DTC, and we then established a 52-point scoring system. Finally, we determined that 10 points was the optimal score for predicting RR-DTC, and the associated AUC was 0.898. The scoring system had a higher predictive value than any other single independent predictor. Its sensitivity, specificity, and Youden index values were 76.0%, 93.0%, and 0.69, respectively, at 10 points. RR-DTC and non-RR-DTC were determined with 10 cutoff values. Subsequently, 169 individuals were correctly classified as RR-DTC, 168 individuals were correctly classified as non-RR-DTC, 13 individuals were misclassified as RR-DTC, and 54 individuals were misclassified as non-RR-DTC, with an accuracy of 83.41%. Subsequently, the visual prediction RR-DTC nomogram model constructed in this study had a c-Index of 0.9. The first behavioral score scale calculates the corresponding score of each risk factor according to the situation of each patient, and then add the scores to obtain the total score. Then, the corresponding score point was found in the total score scale, and the corresponding probability scale coordinate below the vertical was the probability of suffering from RR-DTC.

There have been many studies on the prognostic factors associated with RR-DTC; however, the identification of predictors of RR-DTC rather than pure prognostic factors may be helpful in changing prognostic strategies and outcomes [13]. In a previous study, Li et al. explored RR-DTC predictors and found that certain factors were highly correlated with RR-DTC, including smoking, tumor type, extrathyroid extension, pN stage, and number and rate of lymph node metastasis [12]. However, in view of the latest advances in genetic analysis of thyroid tumors, including molecular methods, we have incorporated molecular imaging characteristics into our prediction model for RR-DTC.

In our study, 55.2% (223/404) of patients developed RR-DTC, and 28.7% (116/404) had distant metastasis, which was consistent with the literature reporting that 7–23% of patients with DTC had distant metastasis [14]. However, 77.6% (90/116) of patients with distant metastases developed RR-DTC in our study, which was higher than the corresponding rate of a previous work showing that 25–50% of patients with distant metastases developed RR-DTC [15]. One explanation is that our study only comprised patients with metastatic DTC who had undergone PET/CT imaging, the use of which depends on the judgment of their physician; meanwhile, patient decisions were also an important factor, which may not fully reflect the true clinical features of metastatic DTC. According to Schlumberger et al. [7], even after an adequate stimulation by thyroid-stimulating hormone and in the absence of excess iodine, only two-thirds of patients with metastases show substantial radioactive iodine uptake, and only 42% of them achieve a cure. According to the definition of RR-DTC, the remaining 58% of patients who are not cured would be classified as having RR-DTC, which was similar to the rate observed in our cohort (55.2%). The possible explanation for our high RR-DTC ratio is that lymph node metastasis accounts for the majority (70.5%); some metastatic lymph nodes take iodine the first time and do not take iodine the second time, but the lesions persist, while some metastatic lymph nodes do not take iodine the first time. In addition, there are also patients with multiple sites of metastasis; if one lesion does not receive iodine, patients are also judged to have RR-DTC.

Using univariate analysis, our study found age to be an important factor. Prior studies have shown that the adverse effect of age on prognosis gradually increases with each decade, especially after 40–45 years [16, 17]. Different studies use different cutoff values; in this study, the cutoff age was 48 years. In the Union for International Cancer Control/American Joint Committee on Cancer (UICC/AJCC) staging system, an age threshold of 45 years is one of the main criteria [13]. It is inferred from this that an older age results in a greater possibility of RR-DTC development, leading to an increased risk of death [18]. The cutoff point value of diagnostic age was changed from 45 to 55 years in the eighth edition of the AJCC TNM staging system. Wassermann et al. showed that age ≥ 60 years significantly affected a patient's cancer-specific survival after the detection of RR-DTC [19]. Studies have shown that some elderly men have more advanced disease, lower disease-free survival, and higher mortality than female patients [20, 21]. However, whether sex can predict the occurrence of RR-DTC remains unknown; our results showed that sex was not a statistically significant factor. Operation frequency was another predictive factor. Cervical scar adhesion, unclear anatomical level, recurrent external invasion of residual cancer after the initial operation, and the incidence of complications reduce the possibility of complete resection of the tumor, thus, increasing the risk of RR-DTC. Some studies have suggested that some pathological subtypes of thyroid cancer are more likely to develop into RR-DTC, such as follicular thyroid cancer, Hürthle cell carcinoma, and poorly differentiated thyroid cancer [22, 23]. One meta-analysis also confirmed that the pathological subtype was a predictor of RR-DTC [6]. However, our results showed that histological subtype was not a statistically significant factor, although there was a relatively low proportion of adverse pathological subtypes in our study, which may explain this finding.

Next, we discuss the conclusions of the molecular imaging in RR-DTC. In our study, ^18^F-FDG uptake was a predictor for RR-DTC, whereas iodine uptake was not. This was surprising, as three out of five definitions of RR-DTC require lesions to be RAI negative, but RAI uptake was not a predictor. A possible reason is that although the definition of RR-DTC involves whether iodine is ingested or not, the definition of RR-DTC contains a wide range of contents, and it may become RR-DTC regardless of whether iodine is ingested or not. Therefore, this variable was not selected in the screening. This finding is consistent with the corresponding of a study by Kang et al., who showed that RAI uptake of metastasis was not correlated with RR-DTC, but FDG uptake was negatively correlated with RR-DTC [10]. Previous studies have shown that despite repeated RAI treatment, more than 50% of patients eventually show disease progression and are ultimately considered refractory to RAI [24]. Moreover, more courses of prior TSH stimulation before iodine-131 administration, as well as more administration of probably overused iodine-131, may lead to a higher tumor burden [25–27]. Therefore, they may be of great value in the early identification of patients with RR-DTC and in combination with other therapeutic modalities [27, 28]. Early ^18^F-FDG imaging can predict the occurrence of RR-DTC and help make an accurate prognosis because RR-DTC is closely related to iodine treatment response. When FDG PET is used in conjunction with RAI whole-body scan (WBS), we can obtain the metabolic information of the two radioactive tracers and infer the differentiation status of thyroid carcinoma at the same time. Many studies have shown that regardless of the affinity of iodine-131, FDG uptake is an adverse prognostic factor [10, 29, 30]. Incidentally, thyroglobulin response can also predict RR-DTC [18], but it lags behind molecular imaging, so it was not included in our prediction model. However, the advantage of ^18^F-FDG PET/CT lies in its ability to identify and locate tumor lesions. Furthermore, an SUVmax of 2.5 points was selected as the threshold value for positive PET/CT results in this study. This value was used because it is generally believed that the SUV is more likely to indicate malignant lesions above 2.5–3.0 points, while benign lesions < 2.0 points are likely larger. Between these two values, the benign and malignant nature of the lesion cannot be determined, and the lesion is classified as a suspicious lesion [31]. Therefore, this standard has been selected in many studies in the literature [32–34], and we did the same in our study. In one research [35], it was found that the mean SUVmax of the suspected lesions after performing PET/CT in patients with differentiated thyroid cancer with elevated thyroglobulin levels and negative ^131^I whole-body scan findings was 2.9 ± 4.5 points.

In univariate analysis, some variables could not be used as independent factors of the disease. Therefore, multivariate analysis was conducted, and several scoring systems for predicting disease-specific mortality based on clinical and pathological prognostic factors were developed [13]. This research may help establish a predictive scoring system for RR-DTC that incorporates molecular imaging.

Currently, the diagnosis of RR-DTC takes a long time because it relies on the trend of thyroglobulin (Tg) and WBS after multiple RAI treatments, combined with relevant imaging examination results. If the development of RR-DTC was determined according to the normal procedures, it would require 4–77 months in our study, with a median of 6 months. This study contributes to the literature as, to our knowledge, it is the first to show that the occurrence probability of RR-DTC can be predicted at an early stage, without waiting for ≥ 6 months. After obtaining the patient's history and completing the relevant examination before the first treatment of RAI, the prediction of RR-DTC can be made using our scoring system and nomogram. Several prognostic scoring systems have been developed for predicting disease-specific mortality based on the following clinical and pathological prognostic factors: AGES (Age, tumor Grade, Extent, and Size) [36], AMES (Age, distant Metastases, Extent, and Size of primary tumor) [37], MACIS (distant Metastases, Age, Completeness of surgery, Invasion of extrathyroidal tissues, and Size of the primary tumor) [38], De Groot's Clinical Classification [39], and National Thyroid Cancer Treatment Cooperative Study Classification [40]. Studies on the use of nomograms for thyroid cancer include those determining the prognostic factors of death because of specific and other causes in patients with DTC and those assessing the preoperative diagnosis of sonographically indeterminate/suspicious lymph node metastasis in such patients by ultrasound [41, 42]. However, only a few studies have reported the prediction of RR-DTC by nomogram. We believe that early FDG PET/CT examination can be beneficial to patients with metastatic DTC. First, we can identify as many metastatic lesions as possible. Second, if the patient shows FDG-positive results, we suggest comprehensive intervention as soon as possible. In our study, one patient with multiple site metastasis showed FDG PET/CT-positive findings, as well as positive findings observed on post-RAI WB therapy scintigraphy after iodine treatment. After two sessions of iodine treatment, good outcomes were noted, the solid metastasis tumor was reduced, and the biochemical improvement was obvious. After 2 years, progress began to appear, and treatment with specific drugs was initiated. Unfortunately, this patient has a poor prognosis. If FDG PET/CT results are positive, research will be conducted to ascertain whether early intervention can prolong the time of disease progression. We hope to show that early PET/CT examination can be beneficial for patients in clinical practice. Details of the other three factors (i.e., age at diagnosis, recurrence between the operation and iodine-131 treatment, and the site of metastasis) can be obtained from patient history data and associated imaging evaluation (ultrasound scan of the neck, chest CT, and other conventional imaging) if the patient did not undergo PET/CT examination prior to treatment. These factors also have some clinical significance in predicting RR-DTC in some patients. For example, a postoperative differentiated thyroid cancer patient older than 48 years who had a recurrence of the tumor before the first iodine-131 treatment has > 90% probability of developing RR-DTC in the future. If the current examination also found more than two sites of metastasis, his probability of developing RR-DTC in the future would be > 99%.

Given its retrospective design, this study is inherent to selection bias. Although this was a retrospective study, our patients were actually divided into two categories. Our team recommended PET/CT examination for patients with multiple metastases and significantly elevated Tg levels from 2014 to 2018. In total, 44 out of 404 (10.9%) patients were enrolled in this stage. With improvements in clinical experience and professional knowledge, we recommended PET/CT examination for the following patients since 2019: patients with structural metastases indicated by imaging before RAI treatment; those with unexplained Tg level elevation > 10 ng/mL after DTC and before RAI treatment; or those in whom pre-RAI imaging examination and stimulating Tg did not indicate metastatic tendency, but the RAI suggested the presence of metastatic lesions. Thus, these patients were advised to complete PET/CT within 1 week to determine the prognosis. At this stage, 360 out of 404 (89.1%) patients were enrolled, indicating that our data represent 89% of the patient cohort with metastatic DTC. Therefore, the selection bias offset had limited influence on our results.

This study had some limitations. First, few metastases could be confirmed pathologically because of the clinical limitations; this needs to be addressed when we study tumor metastasis. In this study, approximately 11% of our patients' metastases were pathologically obtained, including lymph node, lung, and bone metastases. Other metastases were diagnosed by comprehensive imaging, serological indicators, clinical symptoms, and follow-up examinations. At future follow-up examinations, we should try our best to obtain the pathology of the metastatic lesions of the patients, and at the same time, to perform genetic testing of the metastatic lesions to guide the comprehensive treatment plan in the future. Second, the findings have not been verified at other hospitals. In this study, the sample curve is based on random sampling of the full sample using the bootstrap method (100 interactions). There is a 90% chance that the prediction will be correct. Patients from other centers can be selected for validation in the future. Thus, further studies should be conducted to examine the extrapolation of the model. Finally, the genetic status was not included at the very beginning of the research. Approximately 52% of the patients in this study underwent genetic testing. As not all patients underwent genetic testing, statistical factors were not included in this experiment. Some studies have shown that molecular markers provide a useful insight into the role of predicting the occurrence of RR-DTC, and this is an area for making future research efforts. In the future, we will conduct a prospective study of molecular markers to predict RR-DTC.

## Conclusions

It remains a challenge for clinicians to identify the radioiodine refractory status of DTC early enough to initiate follow-up treatment at the individual level. This study indicated that age, recurrence between the operation and iodine-131 treatment, the uptake of ^18^F-FDG, and the site of metastasis were independent predictors in predicting RR-DTC. The predictive model based on the four factors demonstrated good identification ability of RR-DTC cases. An index score ≥ 10 points was found to be the best score for predicting RR-DTC. In addition, the nomogram model constructed in this study has good predictive ability and stability for the prevalence of RR-DTC. This model may help establish an active surveillance or appropriate treatment strategy for postoperative cases of RR-DTC undergoing follow-up treatment.

## Data Availability

The datasets generated and/or analyzed during the current study are not publicly available due to data security restrictions but are available from the corresponding author on reasonable request.

## Abbreviations

^18^F-FDG: ^18^F-fluorodeoxyglucose
AUC: Area under the curve
CI: Confidence interval
DTC: Differentiated thyroid carcinoma
OR: Odds ratio
PET/CT: Positron emission tomography/computed tomography
RAI: Radioactive iodine
RR-DTC: Radioiodine refractory differentiated thyroid carcinoma
SD: Standard deviation
c-Index: Concordance index

## References

[1] Lim H, Devesa SS, Sosa JA, Check D, Kitahara CM (2017). Trends in thyroid cancer incidence and mortality in the United States, 1974–2013. JAMA.

[2] Haugen BR, Alexander EK, Bible KC, Doherty GM, Mandel SJ, Nikiforov YE (2016). 2015 American Thyroid Association management guidelines for adult patients with thyroid nodules and differentiated thyroid cancer: The American Thyroid Association Guidelines Task Force on Thyroid Nodules and Differentiated Thyroid Cancer. Thyroid.

[3] Cooper DS, Doherty GM, Haugen BR, Kloos RT, Lee SL (2009). Revised American Thyroid Association management guidelines for patients with thyroid nodules and differentiated thyroid cancer: the American Thyroid Association (ATA) guidelines taskforce on thyroid nodules and differentiated thyroid cancer. Thyroid.

[4] Durante C, Haddy N, Baudin E, Leboulleux S, Hartl D, Travagli JP (2006). Long-term outcome of 444 patients with distant metastases from papillary and follicular thyroid carcinoma: benefits and limits of radioiodine therapy. J Clin Endocrinol Metab.

[5] Liu FH, Kuo SF, Hsueh C, Chao TC, Lin JD (2015). Postoperative recurrence of papillary thyroid carcinoma with lymph node metastasis. J Surg Oncol.

[6] Luo Y, Jiang H, Xu W, Wang X, Ma B, Liao T (2020). Clinical, pathological, and molecular characteristics correlating to the occurrence of radioiodine refractory differentiated thyroid carcinoma: a systematic review and meta-analysis. Front Oncol.

[7] Schlumberger M, Brose M, Elisei R, Leboulleux S, Luster M, Pitoia F (2014). Definition and management of radioactive iodine-refractory differentiated thyroid cancer. Lancet Diabetes Endocrinol.

[8] Jin Y, Van Nostrand D, Cheng L, Liu M, Chen L (2018). Radioiodine refractory differentiated thyroid cancer. Crit Rev Oncol Hematol.

[9] Cooray SD, Topliss DJ (2017). The management of metastatic radioiodine-refractory differentiated thyroid cancer requires an integrated approach including both directed and systemic therapies. Endocrinol Diabetes Metab Case Rep.

[10] Kang SY, Bang JI, Kang KW, Lee HY, Chung JK (2019). FDG PET/CT for the early prediction of RAI therapy response in patients with metastatic differentiated thyroid carcinoma. PLoS ONE.

[11] Manohar PM, Beesley LJ, Bellile EL, Worden FP, Avram AM (2018). Prognostic value of FDG-PET/CT metabolic parameters in metastatic radioiodine-refractory differentiated thyroid cancer. Clin Nucl Med.

[12] Li G, Lei J, Song L, Jiang K, Wei T, Li Z (2018). Radioiodine refractoriness score: a multivariable prediction model for postoperative radioiodine-refractory differentiated thyroid carcinomas. Cancer Med.

[13] Soares P, Celestino R, Melo M, Fonseca E, Sobrinho-Simões M (2014). Prognostic biomarkers in thyroid cancer. Virchows Arch.

[14] Vaisman F, Carvalho DP, Vaisman M (2015). A new appraisal of iodine refractory thyroid cancer. Endocr Relat Cancer.

[15] Anderson RT, Linnehan JE, Tongbram V, Keating K, Wirth LJ (2013). Clinical, safety, and economic evidence in radioactive iodine-refractory differentiated thyroid cancer: a systematic literature review. Thyroid.

[16] Hay ID, Thompson GB, Grant CS, Bergstralh EJ, Dvorak CE, Gorman CA (2002). Papillary thyroid carcinoma managed at the Mayo Clinic during six decades (1940–1999): Temporal trends in initial therapy and long-term outcome in 2444 consecutively treated patients. World J Surg.

[17] Russell MA, Gilbert EF, Jaeschke WF (1975). Prognostic features of thyroid cancer. A long-term followup of 68 cases. Cancer.

[18] Wang C, Zhang X, Li H, Li X, Lin Y (2017). Quantitative thyroglobulin response to radioactive iodine treatment in predicting radioactive iodine-refractory thyroid cancer with pulmonary metastasis. PLoS ONE.

[19] Ito Y, Miyauchi A, Ito M, Yabuta T, Masuoka H, Higashiyama T (2014). Prognosis and prognostic factors of differentiated thyroid carcinoma after the appearance of metastasis refractory to radioactive iodine therapy. Endocr J.

[20] Kilfoy BA, Devesa SS, Ward MH, Zhang Y, Rosenberg PS, Holford TR (2009). Gender is an age-specific effect modifier for papillary cancers of the thyroid gland. Cancer Epidemiol Biomark Prev.

[21] Gilliland FD, Hunt WC, Morris DM, Key CR (1997). Prognostic factors for thyroid carcinoma. A population-based study of 15,698 cases from the Surveillance, Epidemiology and End Results (SEER) program 1973–1991. Cancer.

[22] Kim HJ, Lee JI, Kim NK, Min YK, Kim SW, Chung JH (2013). Prognostic implications of radioiodine avidity and serum thyroglobulin in differentiated thyroid carcinoma with distant metastasis. World J Surg.

[23] Ghossein RA, Hiltzik DH, Carlson DL, Patel S, Shaha A, Shah JP (2006). Prognostic factors of recurrence in encapsulated Hurthle cell carcinoma of the thyroid gland: a clinicopathologic study of 50 cases. Cancer.

[24] Sabra MM, Dominguez JM, Grewal RK, Larson SM, Ghossein RA, Tuttle RM (2013). Clinical outcomes and molecular profile of differentiated thyroid cancers with radioiodine-avid distant metastases. J Clin Endocrinol Metab.

[25] Cheng L, Fu H, Jin Y, Sa R, Chen L (2020). Clinicopathological features predict outcomes in patients with radioiodine-refractory differentiated thyroid cancer treated with sorafenib: a real-world study. Oncologist.

[26] Rowe CW, Paul JW, Gedye C, Tolosa JM, Bendinelli C, McGrath S (2017). Targeting the TSH receptor in thyroid cancer. Endocr Relat Cancer.

[27] Wu D, Gomes Lima CJ, Moreau SL, Kulkarni K, Zeymo A, Burman KD (2019). Improved survival after multimodal approach with ^131^I treatment in patients with bone metastases secondary to differentiated thyroid cancer. Thyroid.

[28] Zhao CL, Qiu ZL, Chen LB, Yuan ZB, Luo QY (2013). Sustained and diffuse 131I avid bone metastases with low thyroglobulin levels in a patient with papillary thyroid carcinoma. Clin Nucl Med.

[29] Deandreis D, Al Ghuzlan A, Leboulleux S, Lacroix L, Garsi JP, Talbot M (2011). Do histological, immunohistochemical, and metabolic (radioiodine and fluorodeoxyglucose uptakes) patterns of metastatic thyroid cancer correlate with patient outcome?. Endocr Relat Cancer.

[30] Wang W, Larson SM, Fazzari M, Tickoo SK, Kolbert K, Sgouros G (2000). Prognostic value of [18F]fluorodeoxyglucose positron emission tomographic scanning in patients with thyroid cancer. J Clin Endocrinol Metab.

[31] Lin Q, Qi Q, Hou S, Chen Z, Jiang N, Zhang L (2021). Application of pet-CT fusion deep learning imaging in precise radiotherapy of thyroid cancer. J Health Eng.

[32] Nakajo M, Jinguji M, Shinaji T, Tani A, Nakabeppu Y, Nakajo M (2019). 18F-FDG-PET/CT features of primary tumours for predicting the risk of recurrence in thyroid cancer after total thyroidectomy: potential usefulness of combination of the SUV-related, volumetric, and heterogeneous texture parameters. Br J Radiol.

[33] Liu J, Liu B, Yu Y, Chao F, Liu Y, Han X (2018). 18F-FDG PET/CT and ultrasonography in differentiated thyroid carcinoma patients with elevated serum levels of antithyroglobulin antibody, negative Tg and whole body (131)I scan. Hell J Nucl Med.

[34] Kim BH, Kim SJ, Kim K, Kim H, Kim SJ, Kim WJ (2015). High metabolic tumor volume and total lesion glycolysis are associated with lateral lymph node metastasis in patients with incidentally detected thyroid carcinoma. Ann Nucl Med.

[35] Na SJ, Yoo IR, Lin C, Lin Q, Kim SH, Chung SK (2012). Diagnostic accuracy of (18)F-fluorodeoxyglucose positron emission tomography/computed tomography in differentiated thyroid cancer patients with elevated thyroglobulin and negative (131)I whole body scan: evaluation by thyroglobulin level. Ann Nucl Med.

[36] Hay ID, Grant CS, Taylor WF, McConahey WM (1987). Ipsilateral lobectomy versus bilateral lobar resection in papillary thyroid carcinoma: a retrospective analysis of surgical outcome using a novel prognostic scoring system. Surgery.

[37] Cady B (1998). Papillary carcinoma of the thyroid gland: treatment based on risk group definition. Surg Oncol Clin N Am.

[38] Hay ID, Bergstralh EJ, Goellner JR, Ebersold JR, Grant CS (1993). Predicting outcome in papillary thyroid carcinoma: Development of a reliable prognostic scoring system in a cohort of 1779 patients surgically treated at one institution during 1940 through 1989. Surgery.

[39] DeGroot LJ, Kaplan EL, McCormick M, Straus FH (1990). Natural history, treatment, and course of papillary thyroid carcinoma. J Clin Endocrinol Metab.

[40] Sherman SI, Brierley JD, Sperling M, Ain KB, Bigos ST, Cooper DS (1998). Prospective multicenter study of thyroiscarcinoma treatment: Initial analysis of staging and outcome. National thyroid Cancer Treatment Cooperative Study Registry Group. Cancer.

[41] Li C, Xu F, Huang Q, Han D, Zheng S, Wu W (2021). Nomograms for differentiated thyroid carcinoma patients based on the eighth AJCC staging and competing risks model. JNCI Cancer Spectr.

[42] Guo Q, Sun C, Chang Q, Wang Y, Chen Y, Wang Q (2022). Contrast-enhanced ultrasound-based nomogram for predicting malignant involvements among sonographically indeterminate/suspicious cervical lymph nodes in patients with differentiated thyroid carcinoma. Ultrasound Med Biol.

